# Efficacy and safety of cryoballoon ablation combined with vein of Marshall ethanol infusion for persistent atrial fibrillation: a comparative cohort study

**DOI:** 10.3389/fcvm.2026.1685822

**Published:** 2026-02-12

**Authors:** Meixiang Wang, Hao Jiang, Xiaohai Jiang, Jianmei Sha, Ming Chu, Zhongbao Ruan

**Affiliations:** Department of Cardiology, The Affiliated Taizhou People’s Hospital of Nanjing Medical University, Taizhou School of Clinical Medicine, Nanjing Medical University, Taizhou, China

**Keywords:** atrial fibrillation, atrial tachyarrhythmia, cryoballoon ablation, left atrial diameter, vein of Marshall ethanol infusion

## Abstract

**Background:**

Ablation strategies for persistent atrial fibrillation (AF) remain controversial. Vein of Marshall ethanol infusion (VOM-EI) has been proposed to improve outcomes of catheter ablation for AF. This study aimed to evaluate the efficacy and safety of combining cryoballoon ablation (CBA) with VOM-EI in patients with persistent AF.

**Methods:**

This was a single-center, real-world, non-randomized cohort study including consecutive patients with persistent AF who underwent CBA with or without VOM-EI between March 2021 and May 2024.Propensity score matching (PSM) and inverse probability of treatment weighting (IPTW) were applied retrospectively to balance baseline characteristics between groups. The primary endpoint was freedom from atrial tachyarrhythmia at 12 months postablation. Safety outcomes included procedural complications and all-cause mortality.

**Results:**

After PSM, 47 patients in the CBA + VOM-EI group and 94 in the CBA-alone group were included in the analysis. At 12 months, freedom from atrial tachyarrhythmia was higher in the CBA combined with VOM-EI group (78.7%) compared with the CBA-alone group [57.4%; hazard ratio (HR): 0.42; 95% confidence interval (CI): 0.20–0.87; *P* = 0.020] in the PSM cohort. A significantly lower risk of recurrent atrial tachyarrhythmia was also observed in the CBA combined with VOM-EI group in the IPTW-weighted cohort (HR: 0.38; 95% CI: 0.18–0.78; *P* = 0.008). Procedural complication rates were similar between groups. Increased left atrial diameter (LAD) and longer AF duration were identified as risk factors of arrhythmia recurrence.

**Conclusion:**

CBA combined with VOM-EI is a feasible, effective, and safe strategy for treating persistent AF, with approximately 80% of patients remaining free from atrial arrhythmia recurrence at 1-year follow-up and a low incidence of complications. In addition, elevated LAD and longer AF duration were considered risk factors for atrial arrhythmia recurrence. These findings necessitate large-scale randomized controlled studies for further validation.

## 1 Introduction

Atrial fibrillation (AF), an independent risk factor for stroke, is the most common sustained arrhythmia encountered in clinical practice, affecting approximately 59 million individuals worldwide (1, 2). AF is a chronic and progressive disorder with remodeling in the structure, architecture, contractility, and electrophysiology of the atria (3). These aforementioned changes drive the progression from paroxysmal AF to sustained AF, such as persistent AF, which often requires medical intervention for termination (4). Catheter ablation, including radiofrequency ablation (RFCA), cryoballoon ablation (CBA), and pulse field ablation, has become a first-line therapy for sinus rhythm control in AF patients, remarkedly reducing the risk of stroke, heart failure, acute coronary syndrome, and cardiovascular mortality (5). Several pivotal studies have shown that CBA, as an alternative to RFCA, is an effective and safe therapy for both paroxysmal and persistent AF (6, 7). Pulmonary vein isolation (PVI) is the cornerstone of catheter ablation for symptomatic paroxysmal AF. Nevertheless, other underlying mechanisms may also play a crucial role in the progression of persistent AF, affecting the maintenance of sinus rhythm following PVI alone (8). Although several catheter ablation strategies for persistent AF have been proposed, such as stepwise ablation, mitral isthmus block, and electrophysiological substrate ablation, no single procedure has demonstrated clear superiority over others (9, 10). Hence, catheter ablation strategies for persistent AF remain controversial.

The vein of Marshall (VOM) contains muscular bundles, nerve fibers, and arrhythmogenic foci and has been implicated in the initiation and maintenance of persistent AF. Recent studies show that VOM ethanol infusion (VOM-EI) is feasible and safe during catheter ablation for AF. The combination of RFCA with VOM-EI has been associated with long-term freedom from atrial arrhythmia in patients with persistent AF (11). Previous literature has demonstrated that CBA combined with VOM-EI is effective and safe compared to RFCA combined with VOM-EI in patients with paroxysmal AF (12). To date, there have been limited studies exploring the feasibility and safety of CBA combined with VOM-EI in patients with persistent atrial fibrillation. Hence, the present study aimed to investigate whether the addition of VOM-EI to CBA improves ablation results in patients with persistent AF.

## 2 Methods

### 2.1 Study population

This was a single-center, real-world, non-randomized cohort study conducted at the Affiliated Taizhou People's Hospital of Nanjing Medical University. Consecutive patients with persistent AF who underwent CBA with or without adjunctive VOM-EI between March 2021 and May 2024 were included. The inclusion criteria were as follows: (1) age between 18 and 85 years; (2) diagnosis of persistent AF (episodes lasting more than 7 days and resistant or intolerant to at least one antiarrhythmic drug); (3) deemed candidates for CBA with or without VOM-EI; and (4) willingness to comply with follow-up requirements. Exclusion criteria included (1) prior AF ablation; (2) left atrial (LA) diameter ≥55 mm measured in the parasternal long-axis view; (3) presence of LA thrombus on cardiac computed tomography angiography (CCTA); (4) thrombocytopenia or contraindications to anticoagulation (e.g., warfarin, heparin, or direct factor Xa inhibitors); (5) severe structural heart diseases (e.g., moderate-to-severe mitral regurgitation, dilated cardiomyopathy, hypertrophic cardiomyopathy, or rheumatic heart disease); (6) thyroid dysfunction; (7) severe hepatic or renal insufficiency; (8) surgery within 90 days; and (9) pregnancy.

Treatment allocation was non-randomized. The decision to attempt adjunctive VOM-EI was made before the procedure through shared decision-making (patient preference/physician choice) and was further influenced by intraprocedural anatomical and technical feasibility. Patients in whom VOM-EI could not be performed were analyzed in the CBA-alone group.

Initially, 204 persistent AF participants were enrolled at our center between March 2021 and May 2024. Patients with a history of catheter ablation for AF (*N* = 27), those aged ≥85 years (*N* = 1), and those with missing follow-up data (*N* = 4) were excluded. Ultimately, 171 patients (60 in the CBA combined with VOM group and 111 in the CBA-alone group) were included in the study. Among patients receiving CBA combined with VOM-EI, 13 patients did not undergo VOM-EI (10 due to missing VOM and three due to procedural failure) and were thereby analyzed in the CBA-alone group. The study complied with the Declaration of Helsinki and was approved by the Human Research Ethics Committee of the Affiliated Taizhou People's Hospital of Nanjing Medical University (KY 2022-082-01). Written informed consent was obtained from all participants.

### 2.2 Periprocedural management

Oral anticoagulants or low-molecular-weight heparin were prescribed for at least 1 month before the procedure. CCTA was conducted within 2 days prior to the procedure to exclude LA thrombus and to facilitate three-dimensional reconstruction of the pulmonary veins. Antiarrhythmic drugs (AADs) were discontinued for at least five half-lives prior to the procedure.

### 2.3 Cryoballoon ablation procedure

CBA was initially performed under local anesthesia by three experienced cardiologists. Following puncture of the left femoral vein, a steerable quadripolar catheter was placed in the coronary sinus (CS). Using a fixed-curve sheath (Swartz™, Abbott, Abbott Park, IL, USA) advanced by a single transseptal puncture, the 28-mm second-generation cryoballoon catheter (Arctic Front Advance™, Medtronic, Minneapolis, MN, USA) was introduced to perform sequential cryoablation of the left superior pulmonary vein, left inferior pulmonary vein, right superior pulmonary vein, and right inferior pulmonary vein. Heparin was used to maintain anticoagulation, with an activated clotting time between 250 and 350 s. Ablation strategies were guided by time to isolation (TTI). If the TTI was less than 60 s, the initial ablation was performed for 180 s, followed by a second consolidation ablation for 120 s. If the TTI was more than 60 s, ablation was terminated, the catheter position was adjusted, and the ablation steps were repeated as above. If the TTI could not be achieved, ablation was performed for 120 s with optimal position of the cryoballoon, and a segmental approach was employed by moving the balloon around the pulmonary vein. Phrenic nerve pacing was continuously monitored during the cryoablation of the right pulmonary vein.

The procedural endpoint was durable PVI confirmed by bidirectional conduction block. Entrance block was confirmed by elimination of pulmonary vein potentials recorded on a circular mapping catheter (Achieve™, Medtronic, Minneapolis, MN, USA), and exit block was confirmed by pacing from within the pulmonary vein without atrial capture. After initial confirmation of PVI, a 30-min waiting period was routinely observed, followed by repeated confirmation of bidirectional block. No acute pulmonary vein reconnection was observed during the waiting period; therefore, no additional freeze applications beyond the prespecified protocol were required. When necessary, cardioversion with 200 J under midazolam sedation was performed to restore sinus rhythm.

### 2.4 VOM-EI process

**Figure 1:**
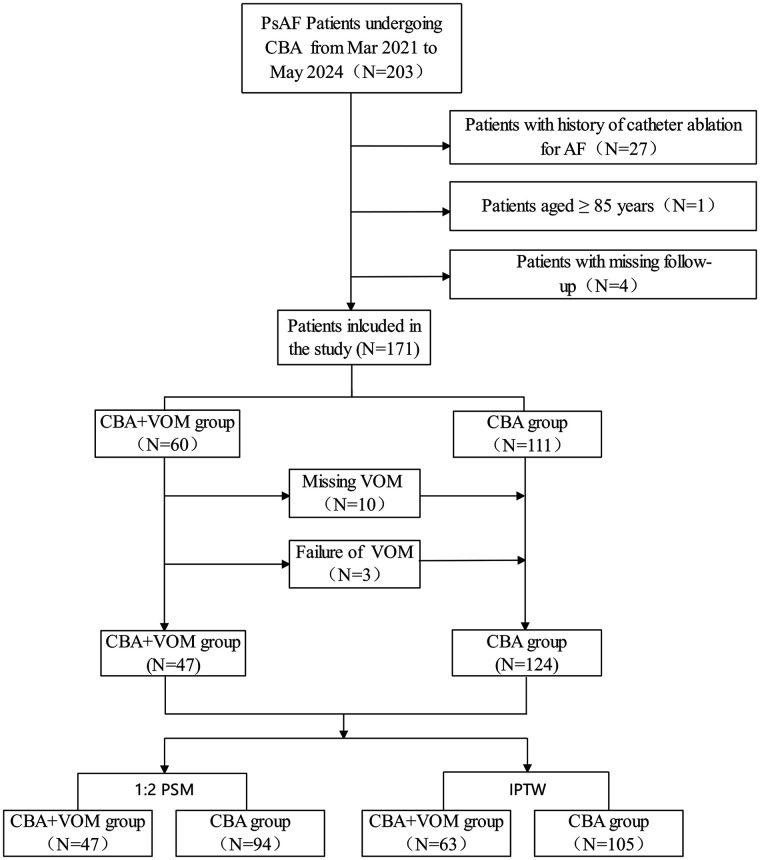
Flowchart of study population. PsAF, persistent AF; CBA, cryoballoon ablation; VOM, vein of Marshall; PSM, propensity score matching; IPTW, inverse probability of treatment weighting.

The VOM-EI process is illustrated in Figure 1. Following local anesthesia, the right femoral vein was punctured, and an 8.5-F long sheath (Swart™ SL1, Medtronic, Minneapolis, MN, USA) was advanced into the lower part of the right atrium. Through this sheath, a 6-F JR4 guiding catheter (Medtronic, Minneapolis, MN, USA) with a 0.014-inch coronary guidewire (Runthrough NS Floppy, Terumo, Tokyo, Japan; 180 cm) was introduced into the CS. The contrast agents were injected through the JR4 catheter to identify the proximal end of the VOM. With the guiding catheter fixed in the position of proximal VOM under fluoroscopic confirmation, the guidewire was advanced to the distal end of the VOM. A 2.0 mm×8 mm angioplasty balloon (Sprinter™ OTW, Medtronic, Minneapolis, MN, USA) was then advanced into the distal end of the VOM along the guidewire. Afterward, the OTW balloon was inflated to a pressure of 6–8 atmospheres. After withdrawal of the guidewire, contrast agents were injected through the balloon lumen to ensure complete occlusion of the VOM. Approximately 2–3 mL of anhydrous ethanol was slowly injected through the OTW balloon. A second same-volume dose of anhydrous ethanol was injected after 2 min. Subsequently, the OTW balloon was withdrawn into the mid and proximal parts of VOM, and the above procedure was repeated. A total of 2–4 injections were performed, with a maximum cumulative volume of anhydrous ethanol not exceeding 12 mL. After the proximal injection of anhydrous ethanol, patients were observed for 10 min to prevent adverse events.

### 2.5 Follow-up

AADs were prescribed to all patients during the blanking period, defined as the first 90 days after the procedure. At our center, amiodarone was routinely prescribed during the blanking period. For patients with contraindications to amiodarone (e.g., thyroid dysfunction or iodine allergy), propafenone was used as an alternative AAD. Beta-blockers were not routinely prescribed for rhythm control as part of the postablation AAD strategy in this cohort. Afterward, discontinuation of AADs was generally considered at the 3-month follow-up in patients without documented recurrence, whereas continuation was individualized based on the overall clinical context. All patients underwent scheduled clinical follow-up visits at 3, 6, and 12 months after the procedure. Regardless of symptoms, a 24-h Holter monitoring was performed at each visit. The primary outcome was freedom from symptomatic or asymptomatic atrial tachyarrhythmias (including atrial tachycardia, atrial flutter, and AF) lasting more than 30 s after the blanking period of the procedure. The primary safety outcome was defined as the occurrence of acute procedural complications or all-cause mortality. Secondary outcomes included time to AF recurrence and rehospitalization for cardiovascular events.

### 2.6 Statistical analysis

The Shapiro–Wilk test was utilized to examine the distribution of continuous variables. Normally distributed continuous variables are presented as means with standard deviations (SD), whereas non-normally distributed continuous variables are presented as medians and interquartile ranges (IQRs). Categorical variables are presented as numbers and proportions. Student’s *t*-test or the Mann–Whitney *U*-test was used to examine the difference between continuous parameters. Pearson’s chi-square test was used to calculate differences between categorical variables.

Given the non-random treatment allocation, propensity score methods were applied to reduce confounding. Propensity scores were estimated to predict the probability of exposure based on observed covariates. These scores were then used to adjust for baseline differences between groups, with the aim of approximating the balance in baseline characteristics and improving the validity of causal inference (13). Propensity scores representing the probability of receiving adjunctive VOM-EI were estimated using a multivariable logistic regression model that included clinically relevant covariates: age, gender, body mass index (BMI), CHA_2_DS_2_-VASc score, HAS-BLED score, smoking status, alcohol use, hypertension, diabetes mellitus (DM), coronary heart disease (CHD), history of cardiac surgery, LA diameter, left ventricular ejection fraction (LVEF), N-terminal probrain natriuretic peptide (NT-proBNP), low-density lipoprotein cholesterol (LDL-C), high-density lipoprotein cholesterol (HDL-C), and total cholesterol (TC). The study conducted propensity score matching (PSM) using a 1:2 ratio between the CBA combined with VOM-EI group and the CBA-alone group to balance clinical characteristics between the two groups. In addition, the inverse probability of treatment weighting (IPTW) method was used to examine the recurrent atrial tachyarrhythmia between the two groups. Covariate balance was assessed using standardized mean differences (SMDs).

Acute procedural and safety outcomes were compared between groups in both the prematched and postmatched cohorts. Specifically, continuous procedural metrics (e.g., ablation time and fluoroscopy time) and categorical procedural variables (e.g., conversion to sinus rhythm) were compared using the tests described above. The incidence of acute procedural complications was compared between groups as a categorical outcome.

The primary outcome (recurrent atrial tachyarrhythmia) was analyzed using Cox proportional hazards models in the original cohort and in the PSM and IPTW cohorts. The proportional hazards assumption was not violated using the Schoenfeld residuals test. Results are presented as hazard ratios (HRs) with 95% confidence interval (CI).

Univariate and multivariate Cox proportional hazards regression analyses were performed to identify risk factors for recurrent atrial tachyarrhythmia. Variables with *P* < 0.20 in the univariate analyses were subsequently entered into the multivariate Cox model to identify independent predictors.

Statistical analyses were completed by R software (version 4.4.1). A two-sided *P* value < 0.05 was considered statistically significant.

## 3 Results

### 3.1 Clinical characteristics

The clinical characteristics of participants in the two groups are presented in Table 1. In the pre-matched sample, a total of 171 patients were enrolled in the study, comprising 124 patients in the CBA-alone group and 47 patients in the CBA combined with VOM-EI group. In the CBA-alone group, the mean age was 69.3 ± 8.4 years, and women accounted for 46.0% of patients. The mean CHA_2_DS_2_-VASc score and mean HAS-BLED score were 3.3 ± 1.5 and 1.8 ± 1.1, respectively. In the CBA combined with VOM-EI group, the mean age was 69.2 ± 7.9 years, women accounted for 44.7% of patients, and the mean CHA_2_DS_2_-VASc score and mean HAS-BLED score were 3.1 ± 1.4 and 1.6 ± 0.9, respectively. AF duration was comparable between the CBA-alone group and the CBA combined with VOM-EI group (24.8 ± 16.6 vs. 23.4 ± 19.9 months, respectively), with no statistically significant difference between groups. Continuation of AADs was observed in 23.4% of patients in the CBA-alone group and 14.9% in the CBA combined with VOM-EI group, with no statistically significant difference between groups. Imbalances with SMDs >0.1 were identified in various clinical characteristics, including BMI, CHA_2_DS_2_-VASc score, HAS-BLED score, smoking status, alcohol use, DM, LAD, NT-proBNP, LDL-C, HDL-C, and TC. After adjusting for clinical characteristics using PSM and IPTW, baseline characteristics were considered well balanced between the CBA-alone group and CBA combined with VOM-EI group.

**Table 1:** Clinical characteristics of participants.

Variable	Prematched				1:2 PSM				IPTW			
	CBA (*n* = 124)	CBA + VOM-EI (*n* = 47)	SMD	*P* value	CBA (*n* = 94)	CBA + VOM-EI (*n* = 47)	SMD	*P* value	CBA (*n* = 105)	CBA + VOM-EI (*n* = 63)	SMD	*P* value
Age (years)	69.3 ± 8.4	69.2 ± 7.9	0.011	0.950	69.0 ± 8.3	69.2 ± 7.9	0.026	0.885	69.2 ± 8.4	68.4 ± 7.7	0.090	0.605
Gender, *n* (%)	57 (46.0)	21 (44.7)	0.026	>0.999	46 (48.9)	21 (45)	0.085	0.766	48 (45.6)	27 (42.3)	0.069	0.710
AF duration (months)	24.8 ± 16.6	23.4 ± 19.9	0.085	0.314	23.8 ± 15.4	23.8 ± 16.7	0.004	0.513	24.2 ± 15.8	22.6 ± 16.4	0.099	0.533
BMI	24.68 ± 2.79	24.36 ± 3.08	0.108	0.521	24.44 ± 2.88	24.36 ± 3.08	0.027	0.879	24.58 ± 2.75	24.77 ± 3.49	0.060	0.789
CHA_2_DS_2_-VASc	3.3 ± 1.5	3.1 ± 1.4	0.120	0.486	3.1 ± 1.4	3.1 ± 1.4	0.022	0.900	3.2 ± 1.5	3.2 ± 1.4	0.024	0.891
HAS-BLED	1.8 ± 1.1	1.6 ± 0.9	0.211	0.243	1.7 ± 1.0	1.6 ± 0.9	0.846	0.539	1.8 ± 1.1	1.7 ± 0.9	0.020	0.913
NYHA, *n* (%)			0.352	0.291			0.078	0.404			0.091	0.694
I	10 (8.1)	7 (14.9)			12 (12.8)	7 (14.9)			10 (9.5)	6 (9.5)		
II	74 (59.7)	30 (63.8)			59 (62.8)	30 (63.8)			64 (61.0)	39 (61.9)		
III	37 (29.8)	10 (21.3)			22 (23.4)	10 (21.3)			29 (27.6)	17 (27.0)		
IV	3 (2.4)	0 (0.0)			1 (1.1)	0 (0.0)			2 (1.9)	1 (1.6)		
Smoking, *n* (%)	97 (78.2)	33 (70.2)	0.184	0.371	68 (72.3)	33 (70.2)	0.047	0.947	80 (76.2)	47 (74.6)	0.033	0.857
Alcohol drinking, *n* (%)	110 (88.7)	40 (85.1)	0.107	0.704	81 (86.2)	40 (85.1)	0.030	>0.999	92 (87.6)	54 (85.6)	0.045	0.811
Comorbidity, *n* (%)												
Hypertension	74 (59.7)	27 (57.4)	0.045	0.928	56 (59.6)	27 (57.4)	0.045	0.952	44 (42.0)	29 (46.0)	0.073	0.700
DM	22 (17.7)	5 (10.6)	0.205	0.367	13 (13.8)	5 (10.6)	0.098	0.789	16 (15.2)	9 (14.3)	0.041	0.851
CHD	26 (21.0)	9 (19.1)	0.045	0.959	17 (18.1)	9 (19.1)	0.027	>0.999	22 (21.0)	12 (19.0)	0.022	0.905
Cardiac surgery history, *n* (%)	2 (1.6)	1 (2.1)	0.038	>0.999	2 (2.1)	1 (2.1)	<0.001	>0.999	3 (2.9)	2 (3.2)	0.008	0.963
LAD (mm)	45.56 ± 4.24	45.11 ± 4.10	0.110	0.525	45.12 ± 4.46	45.11 ± 4.10	0.002	0.989	45.36 ± 4.32	45.04 ± 3.86	0.080	0.655
LVEF (%)	64.19 ± 8.20	61.43 ± 7.31	0.315	0.079	63.02 ± 8.24	61.43 ± 7.31	0.205	0.263	63.29 ± 8.63	62.54 ± 6.55	0.098	0.557
NT-proBNP (pg/mL)	3,538 ± 2,352	3,002 ± 2,621	0.215	0.207	3,052 ± 1,804	3,002 ± 2,621	0.022	0.894	3,391 ± 2,322	31,523 ± 2,545	0.098	0.593
LDL-C (mmol/L)	2.47 ± 0.82	2.25 ± 0.84	0.262	0.132	2.45 ± 0.74	2.25 ± 0.84	0.255	0.147	2.39 ± 0.83	2.33 ± 0.79	0.071	0.689
HDL-C (mmol/L)	1.21 ± 0.39	1.22 ± 0.33	0.043	0.811	1.22 ± 0.40	1.22 ± 0.33	0.009	0.961	1.21 ± 0.3)	1.21 ± 0.28	0.010	0.950
TC (mmol/L)	4.05 ± 1.08	3.99 ± 1.31	0.050	0.767	4.06 ± 0.99	3.99 ± 1.31	0.060	0.727	4.00 ± 1.06	3.94 ± 1.13	0.051	0.771
AAD continuation, *n* (%)	29 (23.4)	7 (14.9)	0.217	0.314	21 (22.3)	7 (14.9)	0.192	0.297	22 (21.0)	10 (15.9)	0.121	0.406

PSM, propensity score matching; IPTW, inverse probability of treatment weighting; CBA, cryoballoon ablation; VOM-EI, vein of Marshall ethanol infusion; SMD, standardized mean difference; AF, atrial fibrillation; BMI, body mass index; NYHA, New York Heart Association; DM, diabetes mellitus; CHD, coronary heart disease; LAD, left atrial diameter; LVEF, left ventricular ejection fraction; NT-proBNP, N-terminal pro-brain natriuretic peptide; LDL-C, low-density lipoprotein cholesterol; HDL-C, high-density lipoprotein cholesterol; TC, total cholesterol; AAD, antiarrhythmic drug.

### 3.2 Primary outcome

Table 2 shows the association between procedure type and recurrent atrial tachyarrhythmia across the three samples. At the end of the 12-month follow-up, 100 (63.3%) patients were identified free from atrial tachyarrhythmia, including 63 (56.8%) in the CBA-alone group and 37 (78.7%) in the CBA combined with VOM-EI group. After the PSM, freedom from atrial tachyarrhythmia was observed in 54 patients (57.4%) in the CBA-alone group and 37 patients (78.7%) in CBA combined with VOM-EI group. In the prematched population, CBA combined with VOM-EI was significantly associated with a decreased risk of recurrent atrial tachyarrhythmia (HR: 0.39; 95% CI: 0.19–0.80, *P* = 0.007). In the PSM sample, CBA combined with VOM-EI was associated with a 58% lower risk of recurrent atrial tachyarrhythmia (HR: 0.42; 95% CI: 0.20–0.87, *P* = 0.020). The efficacy of CBA combined with VOM-EI was consistent in the IPTW sample (HR: 0.38; 95% CI: 0.18–0.78, *P* = 0.008).

**Table 2:** Summary of results of the primary analysis.

Analysis type	HR (95% CI)	*P*-value
Prematched sample		
CBA	Ref.	Ref.
CBA + VOM-EI	0.39 (0.19–0.80)	0.007
1:2 matched sample		
CBA	Ref.	Ref.
CBA + VOM-EI	0.42 (0.20–0.87)	0.020
Inverse probability of treatment weighting		
CBA	Ref.	Ref.
CBA + VOM-EI	0.38 (0.18, 0.78)	0.008

CBA, cryoballoon ablation; VOM-EI, vein of Marshall ethanol infusion.

### 3.3 Acute procedural outcome

Among the 60 patients initially scheduled to receive CBA combined with VOM-EI, VOM-EI was successfully performed in 47 patients (78.3%). Of the 13 unsuccessful procedures, VOM-EI could not be completed in 10 patients due to the absence of a VOM, and cannulation into the VOM was unsuccessful in three patients. The acute procedural outcomes in the prematched sample are displayed in Supplementary Table S1. The total time of CBA and the fluoroscopy time of CBA were not statistically different between the two groups. In the CBA combined with VOM-EI group, the total time of VOM-EI and fluoroscopy time of VOM-EI were 15.91 ± 2.64  and 5.85 ± 1.33 min, respectively. PVI was successfully achieved in all participants in both groups. In addition, no significant differences were observed between the CBA-alone group and the CBA combined with VOM-EI group with respect to conversion to sinus rhythm or conversion to atrial tachycardia. Acute procedural outcomes in the postmatched sample were similar between groups (Supplementary Table S2).

### 3.4 Safety outcome

Supplementary Tables S3, S4 illustrate the safety outcomes after the procedure in the prematched and postmatched samples. In the prematched sample, eight patients in the CBA-alone group and three patients in the CBA combined with VOM-EI group were identified with acute procedural complications, with no statistically significant difference between groups (incidence: 6.5% vs. 6.4%, *P* > 0.999). In the post-matched sample, the incidence of acute procedural complications remained similar between the two groups (7.4% vs. 6.4%, *P* > 0.999). Components of acute procedural complications were similar in both groups and included acute cardiac tamponade, pericardial effusion without pericardiocentesis, hematoma, arteriovenous fistula, and stroke. No deaths occurred during the follow-up period.

### 3.5 Risk factors for recurrent atrial tachyarrhythmia

Univariate and multivariate Cox hazards proportion analyses were conducted to identify potential risk factors for recurrent atrial tachyarrhythmia in both the prematched and postmatched populations. As shown in Supplementary Table S5, LAD was considered a risk factor for recurrent atrial tachyarrhythmia in the univariate analysis (HR: 1.10, 95% CI: 1.03–1.18, *P* = 0.006). Longer AF duration was associated with an increased risk of atrial tachyarrhythmia recurrence in the univariate analysis (HR: 1.03, 95% CI: 1.02–1.04, *P* < 0.001). After adjustment for possible confounders in the multivariate analysis, LAD (HR: 1.08, 95% CI: 1.01–1.16, *P* = 0.041) and AF duration (HR: 1.02, 95% CI: 1.01–1.03, *P* = 0.001) remained risk factors for atrial tachyarrhythmia recurrence. In the postmatched sample, LAD was also associated with recurrent atrial tachyarrhythmia in both univariate and multivariate analyses (Supplementary Table S6). Hence, patients with larger LADs were more likely to suffer from atrial tachyarrhythmia recurrence.

## 4 Discussion

### 4.1 Main findings

The main findings of this comparative cohort study are as follows. First, CBA combined with VOM-EI is a feasible and effective procedure for the treatment of persistent AF, achieving freedom from atrial arrhythmia in approximately 80% of patients at 1-year follow-up postprocedure. Second, CBA combined with VOM-EI is a safe procedure, with rates of acute procedural complications, all-cause mortality, and rehospitalization for cardiovascular events comparable to those of CBA alone. Third, increased LAD and longer AF duration were risk factors for atrial arrhythmia recurrence in patients with persistent AF.

### 4.2 Feasibility CBA combined with VOM-EI

The VOM, identified as a trigger of AF, is a source of spontaneous atrial electrical activity. In addition to serving as an AF trigger, the muscle bundles in the VOM may play a critical role in the development of macro-re-entrant perimitral flutter, which is the most prevalent form of atrial tachycardia observed following AF ablation. Furthermore, the VOM has been recognized as a crucial factor in residual conduction across the mitral isthmus and in the development of left atrial re-entrant tachycardias following AF ablation (14, 15). Various animal studies and clinical studies have demonstrated termination of AF with ablation targeting the VOM (16–18). The VENUS trial, a pivotal randomized controlled trial, investigated the efficacy and safety of RFCA combined with VOM-EI compared with RFCA alone in patients with persistent AF (17). In the VENUS trial, the total mean time of VOM-EI was 42.5 ± 32.8 min, with a mean fluoroscopy time of 11.7 ± 11.0 min. Among patients undergoing RFCA combined with VOM-EI, the success rate was approximately 84%. In our study, the VOM-EI procedure required only 15.91 min on average, with a mean fluoroscopy time of 5.85 min. Among patients undergoing CBA combined with VOM-EI, successful completion of the VOM-EI procedure was achieved in 78.3%. The most common reason for VOM-EI procedural failure was the absence of a VOM, accounting for 76.9% of unsuccessful cases. Unsuccessful cannulation accounted for approximately one-quarter of failures. Hence, CBA-combined VOM-EI emerged as a feasible procedure in our study, with high success rate and a short procedure duration.

### 4.3 Efficacy of CBA combined VOM-EI

Despite relatively limited evidence, recent studies have shown that CBA achieves freedom from atrial tachyarrhythmia comparable to other ablation strategies, with a lower incidence of cardiac tamponade in patients with persistent AF (19, 20). A systemic review encompassing 212 studies of persistent AF ablation included 1,028 patients undergoing catheter ablation combined with VOM-EI and 1,605 undergoing catheter ablation alone. The study demonstrated a benefit of VOM-EI in reducing atrial tachyarrhythmia recurrence compared to ablation alone [relative risk (RR) 0.70, 95% CI 0.53–0.91]. In our study, CBA alone was selected as the control group, which strengthened the robustness of the findings. PVI was successfully achieved in all patients. At the end of 1-year follow-up, 78.7% of patients in the CBA combined with VOM-EI group and 57.4% in the CBA-alone group were free from recurrent atrial arrhythmia. Multivariate analysis showed that CBA combined with VOM-EI was associated with a 54% lower risk of atrial tachyarrhythmia recurrence compared with CBA alone. This significant difference between the two groups was further ascertained by the log-rank test of the Kalpan–Meier curve. CBA combined with VOM-EI was associated with a lower risk of atrial tachyarrhythmia recurrence compared with CBA alone, as demonstrated by the log-rank test of the Kalpan–Meier curve. These findings suggest that CBA combined with VOM provides effective postprocedural rhythm control.

### 4.4 Safety of CBA combined with VOM-EI

CBA, which can serve as initial treatment for AF, has been a safe ablation strategy, with a short procedure duration and a low incidence of procedural complications (21). Several pivotal randomized controlled studies have demonstrated similar rates of postprocedure adverse events between CBA and RFCA (6, 22, 23). In the STOP AF first trial, the incidence of composite procedural adverse events was as low as 1.9% during the 12-month follow-up (23). Two observational studies have determined the safety of catheter ablation combined with VOM-EI (24, 25). Complications within 30 days were identified in 3.2% patients, with pericardial effusion without pericardiocentesis being the most common complication (25). Another study reported mild pericardial effusion without pericardiocentesis in 10.5% of patients undergoing VOM-EI, and only one patient required drainage for pericardial effusion within 1 year (26). In our study, the incidence of acute procedural complications was low in both the CBA combined with VOM-EI group and the CBA-alone group (7.5% vs. 6.4%, *P* > 0.999). During the 12-month follow-up, no all-cause mortality was observed in either group, and rehospitalization for cardiovascular events was uncommon (4.3% in the CBA combined with VOM-EI group vs. 5.3% in the CBA-alone group). Findings regarding acute and long-term postprocedural complications suggest that CBA combined with VOM-EI was a safe ablation strategy with a low incidence of adverse events.

### 4.5 Risk factor for recurrence of atrial tachyarrhythmia

In our study, elevated LAD was identified as a risk factor for postprocedural atrial tachyarrhythmia recurrence. LAD is measured at end-ventricular systole, when the LA chamber reaches its maximum dimension. Based on guidelines, normal LAD ranges from 27 to 38 mm in women and 30 to 40 mm in men, with LAD exceeding 38 mm in women or 40 mm in men classified as enlarged (27). In our study, the mean LAD was approximately 45 mm in the two groups. Therefore, LAD in our study should be considered elevated. Recent studies have recognized AF as a manifestation of atrial cardiomyopathy, which is associated with various pathophysiological manifestations, including electrical disturbances, mechanical dysfunctions, and endothelial dysfunctions (28). Structural remodeling of the LA and left ventricle may occur during the onset and progression of AF. Elevated LAD indicated atrial remodeling, providing a substrate for atrial tachyarrhythmia occurrence (29). A retrospective study investigated the predictors of AF recurrence after catheter ablation. LAD emerged as an independent predictor after adjustment for possible covariates (HR 1.089, 95% CI 1.036–1.146) (30). Similarly, elevated LAD was associated with an 8% risk of atrial tachyarrhythmia recurrence in our study. This finding indicated that patients with a larger LAD had a higher risk for atrial tachyarrhythmia recurrence, with more concerns raised.

### 4.6 Limitations

Several limitations of this study should be acknowledged. First, this was a cohort study with a relatively small sample size. Therefore, large-scale randomized controlled trials are needed to further validate these findings. Second, the absence of continuous ECG monitoring may have led to underdetection of atrial arrhythmia recurrence. Third, the follow-up duration in the study was limited to 12 months, in line with previous studies (17, 23). Nevertheless, longer-term follow-up, such as 3-year follow-up, is needed to evaluate the efficacy and safety of this approach.

## 5 Conclusions

CBA combined with VOM-EI appears to be a feasible, effective, and safe treatment strategy for persistent AF, with approximately 80% patients remaining free from atrial arrhythmia recurrence at 1-year follow-up and a low incidence of complications. In addition, elevated LAD and longer AF duration were identified as risk factors for atrial arrhythmia recurrence. These findings warrant large-scale randomized controlled studies for further validation.

## Data Availability

The original contributions presented in the study are included in the article/Supplementary Material, further inquiries can be directed to the corresponding authors.

## References

[1] Roth GA Mensah GA Johnson CO Addolorato G Ammirati E Baddour LM Global burden of cardiovascular diseases and risk factors, 1990–2019: update from the GBD 2019 study. J Am Coll Cardiol. (2020) 76(25):2982–3021. 10.1016/j.jacc.2020.11.010 33309175 PMC7755038

[2] Escudero-Martinez I Morales-Caba L Segura T. Atrial fibrillation and stroke: a review and new insights. Trends Cardiovasc Med. (2023) 33(1):23–9. 10.1016/j.tcm.2021.12.001 34890796

[3] Goette A Kalman JM Aguinaga L Akar J Cabrera JA Chen SA EHRA/HRS/APHRS/SOLAECE expert consensus on atrial cardiomyopathies: definition, characterization, and clinical implication. Europace. (2016) 18(10):1455–90. 10.1093/europace/euw161 27402624 PMC6392440

[4] De With RR Erkuner O Rienstra M Nguyen BO Korver FWJ Linz D Temporal patterns and short-term progression of paroxysmal atrial fibrillation: data from RACE V. Europace. (2020) 22(8):1162–72. 10.1093/europace/euaa123 32642768 PMC7400474

[5] Kirchhof P Camm AJ Goette A Brandes A Eckardt L Elvan A Early rhythm-control therapy in patients with atrial fibrillation. N Engl J Med. (2020) 383(14):1305–16. 10.1056/NEJMoa2019422 32865375

[6] Kuck KH Brugada J Fürnkranz A Metzner A Ouyang F Chun KR Cryoballoon or radiofrequency ablation for paroxysmal atrial fibrillation. N Engl J Med. (2016) 374(23):2235–45. 10.1056/NEJMoa1602014 27042964

[7] Su WW Reddy VY Bhasin K Champagne J Sangrigoli RM Braegelmann KM Cryoballoon ablation of pulmonary veins for persistent atrial fibrillation: results from the multicenter STOP persistent AF trial. Heart Rhythm. (2020) 17(11):1841–7. 10.1016/j.hrthm.2020.06.020 32590151

[8] Santangeli P Zado ES Hutchinson MD Riley MP Lin D Frankel DS Prevalence and distribution of focal triggers in persistent and long-standing persistent atrial fibrillation. Heart Rhythm. (2016) 13(2):374–82. 10.1016/j.hrthm.2015.10.023 26477712

[9] Dong JZ Sang CH Yu RH Long DY Tang RB Jiang CX Prospective randomized comparison between a fixed ‘2C3L’ approach vs. Stepwise approach for catheter ablation of persistent atrial fibrillation. Europace. (2015) 17(12):1798–806. 10.1093/europace/euv067 25957039

[10] Yang G Zheng L Jiang C Fan J Liu X Zhan X Circumferential pulmonary vein isolation plus low-voltage area modification in persistent atrial fibrillation: the STABLE-SR-II trial. JACC Clin Electrophysiol. (2022) 8(7):882–91. 10.1016/j.jacep.2022.03.012 35863814

[11] Derval N Duchateau J Denis A Ramirez FD Mahida S Andre C Marshall bundle elimination, pulmonary vein isolation, and line completion for ANatomical ablation of persistent atrial fibrillation (Marshall-PLAN): prospective, single-center study. Heart Rhythm. (2021) 18(4):529–37. 10.1016/j.hrthm.2020.12.023 33383226

[12] Okishige K Kawaguchi N Iwai S Yamauchi Y Keida T Sasano T Comparative study of cryoballoon versus radiofrequency for pulmonary vein isolation when combined with vein of Marshall ethanol infusion for paroxysmal atrial fibrillation. J Atr Fibrillation. (2020) 12(5):2253. 10.4022/jafib.2253 32435354 PMC7237087

[13] Haukoos JS Lewis RJ. The propensity score. JAMA. (2015) 314(15):1637–8. 10.1001/jama.2015.13480 26501539 PMC4866501

[14] Chik WW Chan JK Ross DL Wagstaff J Kizana E Thiagalingam A Atrial tachycardias utilizing the ligament of Marshall region following single ring pulmonary vein isolation for atrial fibrillation. Pacing Clin Electrophysiol. (2014) 37(9):1149–58. 10.1111/pace.12423 24831656

[15] Hwang C Chen PS. Ligament of Marshall: why it is important for atrial fibrillation ablation. Heart Rhythm. (2009) 6(12 Suppl):S35–40. 10.1016/j.hrthm.2009.08.034 19959141

[16] Huang L Gao M Lai Y Guo Q Li S Li C The adjunctive effect for left pulmonary vein isolation of vein of Marshall ethanol infusion in persistent atrial fibrillation. Europace. (2023) 25(2):441–9. 10.1093/europace/euac219 36504017 PMC9935035

[17] Valderrabano M Peterson LE Swarup V Schurmann PA Makkar A Doshi RN Effect of catheter ablation with vein of Marshall ethanol infusion vs catheter ablation alone on persistent atrial fibrillation: the VENUS randomized clinical trial. JAMA. (2020) 324(16):1620–8. 10.1001/jama.2020.16195 33107945 PMC7592031

[18] Hwang C Wu TJ Doshi RN Peter CT Chen PS. Vein of Marshall cannulation for the analysis of electrical activity in patients with focal atrial fibrillation. Circulation. (2000) 101(13):1503–5. 10.1161/01.CIR.101.13.1503 10747341

[19] Kobori A Sasaki Y Pak M Ishikura M Murai R Okada T Comparison of cryoballoon and contact force-sensing radiofrequency ablation for persistent atrial fibrillation in clinical practice. Circ J. (2022) 86(2):290–8. 10.1253/circj.CJ-21-0608 34565782

[20] Shi LB Rossvoll O Tande P Schuster P Solheim E Chen J Cryoballoon vs. radiofrequency catheter ablation: insights from NOrwegian randomized study of PERSistent Atrial Fibrillation (NO-PERSAF study). Europace. (2022) 24(2):226–33. 10.1093/europace/euab281 35134151 PMC8824490

[21] Andrade JG Wazni OM Kuniss M Hawkins NM Deyell MW Chierchia GB Cryoballoon ablation as initial treatment for atrial fibrillation: JACC state-of-the-art review. J Am Coll Cardiol. (2021) 78(9):914–30. 10.1016/j.jacc.2021.06.038 34446164

[22] Andrade JG Champagne J Dubuc M Deyell MW Verma A Macle L Cryoballoon or radiofrequency ablation for atrial fibrillation assessed by continuous monitoring: a randomized clinical trial. Circulation. (2019) 140(22):1779–88. 10.1161/CIRCULATIONAHA.119.042622 31630538

[23] Wazni OM Dandamudi G Sood N Hoyt R Tyler J Durrani S Cryoballoon ablation as initial therapy for atrial fibrillation. N Engl J Med. (2021) 384(4):316–24. 10.1056/NEJMoa2029554 33197158

[24] Sousonis V Combes S Pinon P Combes N Cardin C Zeriouh S A novel stepwise approach incorporating ethanol infusion in the vein of Marshall for the ablation of persistent atrial fibrillation. Front Cardiovasc Med. (2023) 10:1194687. 10.3389/fcvm.2023.1194687 37304968 PMC10251404

[25] Luo T Chen Y Xiong X Cheng G Deng C Zhang J Efficacy and safety of the vein of Marshall ethanol infusion with radiofrequency catheter ablation for the treatment of persistent atrial fibrillation in elderly patients. Front Cardiovasc Med. (2023) 10:1276317. 10.3389/fcvm.2023.1276317 38130690 PMC10733440

[26] Hayasaka K Sasaki T Shirai Y Shimosato H Tahara T Segami S A novel catheter ablation strategy for non-paroxysmal atrial fibrillation combining cryoballoon, radiofrequency, and Marshall-vein ethanol ablations. Pacing Clin Electrophysiol. (2023) 46(6):475–86. 10.1111/pace.14709 37129189

[27] Lang RM Badano LP Mor-Avi V Afilalo J Armstrong A Ernande L Recommendations for cardiac chamber quantification by echocardiography in adults: an update from the American Society of Echocardiography and the European Association of Cardiovascular Imaging. J Am Soc Echocardiogr. (2015) 28(1):1–39.e14. 10.1016/j.echo.2014.10.003 25559473

[28] Goette A Corradi D Dobrev D Aguinaga L Cabrera JA Chugh SS Atrial cardiomyopathy revisited-evolution of a concept: a clinical consensus statement of the European Heart Rhythm Association (EHRA) of the ESC, the Heart Rhythm Society (HRS), the Asian Pacific Heart Rhythm Society (APHRS), and the Latin American Heart Rhythm Society (LAHRS). Europace. (2024) 26(9):euae204. 10.1093/europace/euae204 39077825 PMC11431804

[29] Krezowski JT Wilson BD McGann CJ Marrouche NF Akoum N. Changes in left ventricular filling parameters following catheter ablation of atrial fibrillation. J Interv Card Electrophysiol. (2016) 47(1):83–9. 10.1007/s10840-016-0131-8 27076060

[30] Ding B Liu P Zhang F Hui J He L. Predicting values of neutrophil-to-lymphocyte ratio (NLR), high-sensitivity C-reactive protein (hs-CRP), and left atrial diameter (LAD) in patients with nonvalvular atrial fibrillation recurrence after radiofrequency ablation. Med Sci Monit. (2022) 28:e934569. 10.12659/MSM.934569 35082255 PMC8805343

