# Supplementary material for: Efficacy and safety of cryoballoon ablation combined with vein of Marshall ethanol infusion for persistent atrial fibrillation: a comparative cohort study
Source: Front Cardiovasc Med. 2026 Feb 12;13:1685822. doi: 10.3389/fcvm.2026.1685822 (PMC12935975; doi:10.3389/fcvm.2026.1685822)
Supplement: Supplementary file 1 [file Datasheet1.docx]

**Supplementary Table S1. Procedural outcome of the pre-matched sample.**

|  | CBA (n = 124) | CBA+VOM-EI (n = 47) | P value |
| --- | --- | --- | --- |
| Total time of CBA | 31.88 ± 5.45 | 31.17 ± 5.38 | 0.451 |
| Fluoroscopy time of CBA | 14.65 ± 2.63 | 15.23 ± 3.38 | 0.293 |
| Total time of VOM-EI |  | 15.91 ± 2.64 |  |
| Fluoroscopy time of VOM-EI |  | 5.85 ± 1.33 |  |
| Successful PVI | 111 (100.0) | 47 (100.0) | >0.999 |
| Convert to sinus rhythm | 17 (13.7) | 8 (17.0) | 0.745 |
| Convert to atrial tachycardia | 26 (21.0) | 9 (19.2) | 0.993 |
| Conversion | 82 (66.1) | 30 (63.8) | 0.959 |

CBA, cyroballoon ablation; VOM-EI, vein of Marshall ethanol infusion; PVI, pulmonary vein isolation.

**Supplementary Table S2. Procedural outcome of the matched sample.**

|  | **CBA (n = 94)** | **CBA+VOM-EI (n = 47)** | **P value** |
| --- | --- | --- | --- |
| Total time of CBA | 30.58 ± 5.03 | 31.17 ± 5.38 | 0.841 |
| Fluoroscopy time of CBA | 14.95 ± 3.12 | 15.23 ± 3.38 | 0.689 |
| Total time of VOM-EI |  | 15.91 ± 2.64 |  |
| Fluoroscopy time of VOM-EI |  | 5.85 ± 1.33 |  |
| Successful PVI | 94 (10.00) | 47 (100.0) | >0.999 |
| Convert to sinus rhythm | 13 (13.9) | 8 (17.0) | 0.786 |
| Convert to atrial tachycardia | 19 (20.2) | 9 (19.2) | 0.994 |
| Conversion | 62 (66.0) | 30 (63.8) | 0.958 |

CBA, cyroballoon ablation; VOM-EI, vein of Marshall ethanol infusion; PVI, pulmonary vein isolation.

**Supplementary Table S3. Safety outcome of the procedure in the pre-matched sample.**

|  | CBA  (n = 124) | CBA+VOM-EI  (n = 47) | P value |
| --- | --- | --- | --- |
| Acute procedural complication | 8 (6.5) | 3 (6.4) | >0.999 |
| Acute cardiac tamponade | 1 (0.8) | 0 (0.0) | >0.999 |
| Pericardial effusion without pericardiocentesis | 4 (3.2) | 1 (2.1) | >0.999 |
| Hematoma | 0 (0.0) | 1 (2.1) | 0.657 |
| Arteriovenous fistula | 2 (1.6) | 1 (2.1) | >0.999 |
| Stroke | 1 (0.8) | 0 (0.0) | >0.999 |
| All-cause mortality | 0 (0.0) | 0 (0.0) | >0.999 |
| Rehospitalization for cardiovascular events | 6 (4.8) | 2 (4.3) | >0.999 |

CBA, cyroballoon ablation; VOM-EI, vein of Marshall ethanol infusion.

**Supplementary Table S4. Safety outcome of the procedure in the matched sample.**

|  | CBA  (n = 94) | CBA+VOM-EI  (n = 47) | P value |
| --- | --- | --- | --- |
| Acute procedural complication | 7 (7.4) | 3 (6.4) | >0.999 |
| Acute cardiac tamponade | 1 (1.1) | 0 (0.0) | >0.999 |
| Pericardial effusion without pericardiocentesis | 3 (3.2) | 1 (2.1) | >0.999 |
| Hematoma | 0 (0.0) | 1 (2.1) | 0.657 |
| Arteriovenous fistula | 2 (2.1) | 1 (2.1) | >0.999 |
| Stroke | 1 (1.1) | 0 (0.0) | >0.999 |
| All-cause mortality | 0 (0.0) | 0 (0.0) | >0.999 |
| Rehospitalization for cardiovascular events | 5 (5.3) | 2 (4.3) | >0.999 |

CBA, cyroballoon ablation; VOM-EI, vein of Marshall ethanol infusion.

**Supplementary Table S5. Univariate and multivariate analysis of potential factors for atrial tachyarrhythmia recurrence in the pre-matched sample.**

|  | **Univariate analysis** | | **Multivariate analysis** | |
| --- | --- | --- | --- | --- |
| **Variable** | **HR (95% CI)** | **P value** | **HR (95% CI)** | **P value** |
| Age | 1.00 (0.97 - 1.03) | 0.937 |  |  |
| Female | 1.15 (0.69 - 1.93) | 0.588 |  |  |
| AF duration | 1.03 (1.02 - 1.04) | <0.001 | 1.02 (1.01 - 1.04) | 0.001 |
| BMI | 0.96 (0.88 - 1.06) | 0.419 |  |  |
| CHA_2_DS_2_-VASc | 1.12 (0.94 - 1.34) | 0.196 | 1.05 (0.87 - 1.26) | 0.619 |
| HAS-BLED | 0.96 (0.75 - 1.23) | 0.725 |  |  |
| Smoking | 0.88 (0.46 - 1.66) | 0.687 |  |  |
| Alcohol drinking | 1.19 (0.59 - 2.43) | 0.624 |  |  |
| Hypertension | 0.77 (0.46 - 1.30) | 0.328 |  |  |
| DM | 0.98 (0.47 - 2.08) | 0.967 |  |  |
| CHD | 0.71 (0.35 - 1.45) | 0.346 |  |  |
| Cardiac surgery history | 1.01 (0.14 - 7.33) | 0.989 |  |  |
| NT-proBNP | 1.00 (1.00 - 1.00) | 0.297 |  |  |
| TC | 0.95 (0.75 - 1.20) | 0.638 |  |  |
| LDL-C | 0.76 (0.55 - 1.06) | 0.108 | 0.76 (0.54 - 1.05) | 0.096 |
| HDL-C | 2.25 (1.27 - 4.00) | 0.006 | 1.70 (0.94 - 3.08) | 0.080 |
| LAD | 1.10 (1.03 - 1.18) | 0.006 | 1.08 (1.01 - 1.16) | 0.041 |
| LVEF | 1.03 (1.00 - 1.07) | 0.074 | 1.03 (0.99 - 1.07) | 0.115 |
| Convert to sinus rhythm | 1.54 (0.80 - 2.98) | 0.196 | 1.19 (0.67 - 2.13) | 0.548 |
| Convert to AT | 1.21 (0.65 - 2.24) | 0.551 |  |  |
| Conversion | 0.68 (0.40 - 1.15) | 0.151 | 0.73 (0.38 - 1.37) | 0.279 |

AF, atrial fibrillation; BMI, body mass index; DM, diabetes mellitus; CHD, coronary heart disease; NT-proBNP, N-terminal pro–brain natriuretic peptide; TC, total cholesterol; LDL-C, low-density lipoprotein cholesterol; HDL-C, high-density lipoprotein cholesterol; LAD, left atrial diameter; LVEF, left ventricular ejection fraction; AT, atrial tachycardia.

**Supplementary Table S6. Univariate and multivariate analysis of potential factors for atrial tachyarrhythmia recurrence in the matched sample.**

|  | **Univariate analysis** | | **Multivariate analysis** | |
| --- | --- | --- | --- | --- |
| **Variable** | **HR (95% CI)** | **P value** | **HR (95% CI)** | **P value** |
| Age | 1.00 (0.96 - 1.03) | 0.835 |  |  |
| Female | 1.11 (0.64 - 1.93) | 0.717 |  |  |
| AF duration | 1.03 (1.02 - 1.04) | <0.001 | 1.02 (1.01 - 1.04) | 0.001 |
| BMI | 0.96 (0.87 - 1.06) | 0.441 |  |  |
| CHA_2_DS_2_-VASc | 1.12 (0.95 - 1.33) | 0.186 | 1.05 (0.87-1.26) | 0.619 |
| HAS-BLED | 0.92 (0.73 - 1.17) | 0.521 |  |  |
| Smoking | 0.98 (0.55 - 1.74) | 0.935 |  |  |
| Alcohol drinking | 0.85 (0.42 - 1.72) | 0.653 |  |  |
| Hypertension | 1.45 (0.83 - 2.53) | 0.190 | 1.43 (0.79 - 2.56) | 0.235 |
| DM | 1.38 (0.55 - 3.48) | 0.494 |  |  |
| CHD | 1.21 (0.57 - 2.59) | 0.615 |  |  |
| Cardiac surgery history | 0.70 (0.10 - 5.10) | 0.728 |  |  |
| NT-proBNP | 1.00 (1.00 - 1.00) | 0.213 |  |  |
| LDL-C | 0.72 (0.53 - 1.00) | 0.049 | 0.76 (0.54 - 1.05) | 0.096 |
| HDL-C | 1.88 (0.93 - 3.82) | 0.080 | 1.70 (0.94 - 3.08) | 0.080 |
| TC | 1.02 (0.80 - 1.29) | 0.880 |  |  |
| LAD | 1.10 (1.03 - 1.18) | 0.006 | 1.08 (1.00 - 1.16) | 0.041 |
| LVEF | 1.03 (0.99 - 1.06) | 0.165 | 1.03 (0.99 - 1.07) | 0.115 |
| Convert to sinus rhythm | 1.52 (0.74 - 3.13) | 0.258 |  |  |
| Convert to AT | 0.90 (0.44 - 1.84) | 0.767 |  |  |
| Conversion | 1.00 (0.59 - 1.69) | 0.990 |  |  |

AF, atrial fibrillation; BMI, body mass index; DM, diabetes mellitus; CHD, coronary heart disease; NT-proBNP, N-terminal pro–brain natriuretic peptide; TC, total cholesterol; LDL-C, low-density lipoprotein cholesterol; HDL-C, high-density lipoprotein cholesterol; LAD, left atrial diameter; LVEF, left ventricular ejection fraction; AT, atrial tachycardia.
